# Behavior Classification of Cattle in a Virtual Fencing System Using Tri-Axial Accelerometers and Machine Learning

**DOI:** 10.3390/ani16132022

**Published:** 2026-07-02

**Authors:** Silje Marquardsen Lund, Cino Pertoldi, John Frikke, Christian Sonne, Aage Kristian Olsen Alstrup

**Affiliations:** 1 Department of Chemistry and Bioscience, Aalborg University, Frederik Bajers Vej 7H, 9220 Aalborg, Denmarkcp@bio.aau.dk (C.P.); 2 Nationalpark Vadehavet, Havnebyvej 154, 6792 Rømø, Denmark; 3 Department of Ecoscience, Arctic Research Cluster (ARC), Aarhus University, Frederiksborgvej 399, 4000 Roskilde, Denmark; 4 Department of Nuclear Medicine, Aarhus University Hospital, Palle Juul-Jensens Boulevard 165, 8200 Aarhus, Denmark; 5 Department of Clinical Medicine, Aarhus University, Palle Juul-Jensens Boulevard 11, 8200 Aarhus, Denmark

**Keywords:** virtual fencing, cattle behavior, accelerometer, machine learning, behavior classification, *Bos taurus*

## Abstract

Virtual fencing is increasingly used in grazing systems as a flexible alternative to physical fencing, yet detailed assessments of cattle behavior within such systems remain limited. This study investigates the use of collar-mounted tri-axial accelerometers combined with supervised machine learning to characterize cattle behavior in a virtual fencing system. Seven free-ranging Angus cattle were monitored using accelerometers mounted on a virtual fencing system, GNSS positioning, and virtual fence warning logs. A random forest classifier was developed and trained to identify key behaviors (grazing/feeding, ruminating, lying, standing and locomotion) using features derived from tri-axial accelerometer data. The model achieved high classification performance for grazing/feeding, ruminating, and lying (mean accuracy = 0.87, range = 0.83–0.90), enabling estimation of individual behavioral time budgets. Daily activity patterns were generally stable over time and across individuals. Spatial analyses revealed significant differences in behavior between areas near the virtual fence boundary and interior pasture locations, with increased grazing and reduced ruminating near the boundary, potentially reflecting spatial variation in habitat type or forage availability. In the virtual fencing system, cattle are equipped with collars that emit an auditory warning when they approach a virtual boundary, followed by a low-energy electrical impulse when the warning is ignored over a directional distance of 5–10 m. Event-based analyses showed no consistent short-term changes in either movement intensity and direction nor locomotion following auditory warning events, indicating that cattle habituated to the system did not exhibit uniform behavioral disturbance in response to warnings. These results suggest that accelerometer-based behavior classification can provide fine-scale, non-invasive insights into spatio-temporal cattle behavior in virtual fencing systems. The finding indicates that, in a habituated herd, virtual fencing was not associated with pronounced disruption to the measured behavioral patterns, while highlighting the potential of embedded sensor data for animal-based behavioral monitoring.

## Simple Summary

Virtual fencing is a flexible alternative to physical fencing of cattle. In this study, seven Angus cattle were monitored using accelerometers mounted on a virtual fencing system. The aim of the study was to determine whether sensor data combined with machine learning could be used to identify common cattle behavior such as grazing, resting, lying, standing and walking, and to examine whether the virtual fence influenced cattle behavior. Our findings indicate that, in habituated cattle, virtual fencing was not associated with pronounced disruption of the measured behavioral patterns. This study also highlights the potential of data from animal-mounted sensors for animal-based behavioral monitoring.

## 1. Introduction

In recent years, there has been increasing interest in innovative and technology-driven solutions for sustainable management of cattle [1,2,3,4]. At the same time, attention has increasingly focused on animal welfare within such systems, emphasizing the need for reliable, animal-based welfare assessments rather than sole management- or resource-based indicators [2,5,6]. One emerging technology is virtual fencing with cattle wearing individual collars programmed to respond to virtual boundaries defined by Global Navigation Satellite System (GNSS) coordinates [1,7,8]. As animals approach a virtual boundary, the collar emits an auditory warning, followed by a low-energy electrical impulse if multiple audio warnings are ignored. This system allows animals to associate the auditory cue with the boundary, and adjust their movement accordingly [3,6,7]. Virtual fencing offers a flexible alternative to physical fencing, which can fragment landscapes, restrict wildlife movement, and be practically and financially difficult to adapt to dynamic management needs in conservation grazing and rewilding projects [3,7,8,9,10].

Several studies have demonstrated that cattle habituate well to virtual fencing systems, receiving substantially fewer electrical impulses after an initial learning period [7,8]. Moreover, previous research has generally found no strong indications of compromised welfare associated with virtual fencing, based on measures such as warning frequency, activity levels, and physiological stress indicators, including cortisol concentrations in manure [7,8,10]. However, these assessments have largely relied on indirect or coarse welfare indicators and have provided limited insight into the detailed behavioral responses of the cattle. Animal behavior is widely recognized as a central component of welfare assessment, reflecting how animals interact with their environment and respond to management interventions [5,11,12].

In recent years, accelerometer-based monitoring has become a tool within precision livestock farming, enabling continuous, non-invasive measurement of animal movement and activity [1,11,13,14,15]. Tri-axial accelerometers capture movement along three orthogonal axes and can be used to classify behaviors such as grazing, resting, locomotion and sudden reactions, due to their unique movement patterns [13,14,15,16,17]. When combined with machine learning approaches, accelerometer data allow for automated and fine-scale behavioral classification across long time periods [13,14,18]. Moreover, the combination of accelerometers and GPS-tracking has proven highly effective in mapping both regular behavior and abnormal events, such as sudden escape responses or abnormal movements related to stress [2,13,17]. Here, we investigate beef cattle behavior in a virtual fencing system using tri-axial accelerometer data combined with GNSS positioning. Specifically, we apply a supervised machine learning approach to classify key behaviors and examine (i) daily behavioral time budgets, (ii) spatial differences in behavior relative to the virtual fence boundary, and (iii) short-term behavioral responses to virtual fence warning.

## 2. Materials and Methods

### 2.1. Area and Animals

**Figure 1:**
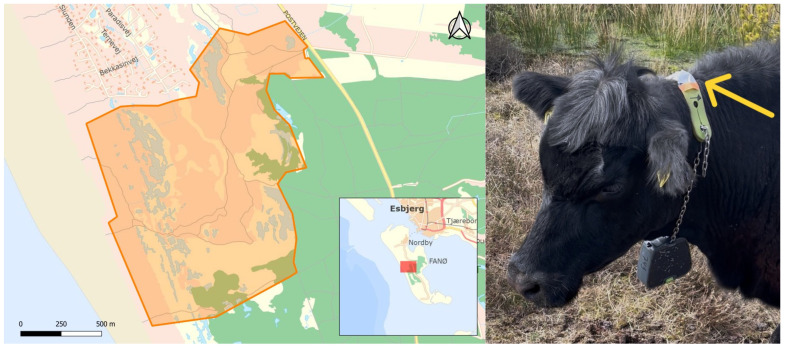
(**Left**) Map showing the pasture (orange marking) on the island of Fanø. All behavioral and spatial analyses were conducted within this pasture, and the virtual fence configuration remained unchanged throughout the study. (**Right**) Example of a Nofence© collar (Molde, Norway) worn by a cow. The arrow indicates the position of the Axivity AX3 three-axis accelerometer (Axivity, York, UK) mounted to the top of the collar using protective wrapping. Photo: Silje Marquardsen Lund.

This study was conducted on a herd of Angus cattle located on the western coast of the island Fanø, Denmark (Figure 1). The area, called “Gåsehullerne”, is a combination of coastal meadow and dune landscape with both heath, wetland, dense pine and spruce forest, and natural ponds and drainage ditches. The forested areas provided natural shade and shelter, and water was available from ponds and ditches within the pasture. The pasture was managed using a virtual fencing system, with stable virtual boundaries throughout the study period. No fences, besides the virtual fence, were present except close to the main island road. The animals were well adapted to both the area and the virtual fence prior to data collection for more than a year.

A total of seven adult Angus cows were included in the study and are referred to as subject A-G throughout the manuscript. The animals constituted the production herd present in the pasture at the time of data collection and were not selected specifically for this study. Six of the individuals calved for the first time after the study period, between 26 May and 30 June 2025, while one cow (subject A) had calved in late summer 2024. All individuals were already equipped with virtual fencing collars as part of an ongoing management and research project evaluating virtual fencing in Denmark, with approval by the Animal Experiments Inspectorate of Denmark. No changes were made to herd composition, grazing management, or animal handling specifically for this study, apart from the attachment of accelerometer sensors to the collars.

### 2.2. Virtual Fence System

At the start of this study, the animals were already equipped with virtual fencing collars. The animals wore Nofence© collars (Figure 1). The collars utilize a system of continuous GNSS signals, alongside sound and electric warnings if the animals cross a virtual boundary. The Nofence© collars were equipped with a battery, two solar panels and an integrated GNSS receiver. The receiver utilizes the GNSS positioning system (GPS and GLONASS), meaning the signals have an uncertainty of 3.5–10 m (GPS accuracy = 3.5–7.8 m, GLONASS accuracy = 5–10 m). The position of the individual animal was updated every 15 min, except when the animal nears the border, where the location needs to be more certain. When an animal approached the virtual boundary, the collar emitted a series of auditory warnings consisting of multiple tones of approximately 82 dB with increasing pitch for 5–20 s, depending on whether the animal changed direction or continued toward the boundary. If the animal continued toward the virtual boundary and was at risk of crossing it, the collar delivered an electric impulse of 0.2 J at 3 kV for one second. If the animal did not respond by slowing down, stopping, or changing direction, additional impulses could be delivered, up to a maximum of three. After the third impulse, the animal is registered as having escaped, and a notification is sent to the owner. The animal’s position continues to be updated, but no further warnings or impulses are delivered.

### 2.3. Accelerometers

Movement was measured with the Axivity AX3 (Axivity, York, UK http://www.axivity.com/product/1 (accessed on 8 April 2025)), a small three-axis accelerometer. Each sensor measures 23.0 mm × 32.5 mm × 7.6 mm, weighs 11 g and is powered by an internal rechargeable battery. The small size and weight compared to cattle body weight over 500 kg ensured that the animals were not disturbed by the extra sensor on the collar. Configuration and data were accessed using the Open Movement (OmGUI) program (https://github.com/openmovementproject/openmovement/wiki/AX3-GUI (accessed on 8 April 2025)). To fit this study, the sampling rate was set to 25 Hz with a sensitivity range of ±8 g; this was chosen on the basis of the review by Riaboff et al. [14] and the length of this study. For collar mounting, sensors were wrapped in cling film and protective mesh. It was then secured to the collar with duct tape (Figure 1) Accelerometer data was collected from 8 April 2025, until 29 April 2025, except for 3 individuals due to loss of the collar-mounted accelerometer in one case and battery failure in two others. One individual (subject G) lost its collar and accelerometer sensor during the study period and two of the batteries in the accelerometers failed and only measured until 11 April 2025 (subject A and B). As a result, one individual was excluded from accelerometer-based analyses, and all subsequent behavioral analyses were therefore based on data from six animals, with reduced data coverage for two individuals.

### 2.4. Behavioral Classification and Video Collection

Behavioral observations used for training and validation of accelerometer-based classification were collected during 8–9 and 11–12 April 2025. Three individuals from the herd were selected at random and the collars were visually marked using colored masking tape to allow reliable identification of individuals during observations. Observations were conducted directly in the field as continuous focal animal observations. To minimize disturbance of the animals, observations were performed from a distance using binoculars. During each observation period, the observer followed the herd and recorded all occurrences and durations of predefined behaviors according to a predefined ethogram (Table 1). If a focal animal was out of sight, no observations were recorded for that individual until it was visible again. Behavioral events were recorded using the Behayve mobile application (IOS v4.4.3, https://www.behayve.com/) To ensure accurate temporal alignment between behavioral observations and accelerometer data, all behavioral recordings were timestamped through the application.

To support validation of the field-based behavioral annotations, video recordings were collected simultaneously using a Canon PowerShot G7 X Mark II camera (Canon Inc., Tokyo, Japan). Video recordings were collected simultaneously during single animal observations, to capture all three individuals when possible. However, due to field conditions and animal movements, this was not always achievable, and video footage did not consistently cover the full duration of observation periods. Video recordings were used exclusively to validate field-based behavioral annotations rather than as a primary source of behavioral data. Video recordings collected during behavioral observations were processed and annotated using BORIS (Behavioral Observation Research Interactive Software, version (v. 9.3.2 2025-04-14)), an open-source event-logging program designed for behavioral studies [19].

The ethogram initially included eight main behavioral categories: grazing/feeding, ruminating, walking, running, standing, lying, sudden movement/reaction to the virtual fence, and other (Table 1). However, due to low occurrence and high variability in movement pattern, such behavior was modified before model training. Specifically, running was merged with walking into a single model class termed “locomotion”, as both behaviors represent locomotion and were difficult to reliably separate based on collar-mounted accelerometer data alone. Sudden movement/reaction to the fence was excluded from the final classification due to its rarity, which limited its suitability for supervised machine learning. Potential short-term responses to virtual fence warnings were therefore assessed using changes in accelerometer-derived movement intensity (VeDBA) and predicted locomotion before and after warning events. The residual “other” category, defined in the ethogram as grooming, drinking, maintenance behavior, and social interactions, was removed due to its high variability, which introduced noise and reduced classification performance. The final set of behaviors used for model development therefore consisted of grazing, ruminating, locomotion, standing, lying.

The total duration of behavioral observations used for the final model training and validation was 22 h, distributed across the three focal animals as follows: subject B = 7.1 h, subject C = 7.2 h, and subject E = 7.7 h.

### 2.5. Model Creation

Accelerometer data was classified using a supervised machine learning approach based on a random forest classifier. All analyses were performed using Python (version 3.13.8; [20]) using standard scientific computing and machine learning libraries, including NumPy (2.3.4), pandas (2.3.3), and scikit-learn (1.7.2), with data processing and model development tailored to three-axis accelerometer data collected at 25 Hz.

#### 2.5.1. Windowing and Segmentation

The continuous accelerometer time series was segmented into fixed length overlapping windows to capture short-term movement patterns. Windows of 5 s (125 samples) were extracted with a 50% overlap between consecutive windows. Each window was assigned a behavioral label based on the dominant behavior present within the window. Only windows containing a single dominant behavior were retained for model training to reduce label noise. Because windows overlapped by 50%, adjacent windows were not statistically independent. Thus, the number of windows represents model input samples rather than independent behavioral observations.

#### 2.5.2. Data Augmentation

To improve model robustness to variation in sensor orientation, data augmentation was applied to the training data. This was considered necessary because collar-mounted sensors may rotate or shift during deployment, causing changes in axis orientation that are unrelated to true behavioral changes. Data augmentation was applied only to the training data. Augmentation was designed to simulate realistic changes in collar positioning and sensor noise without altering the underlying behavioral signal. These included random sign inversion of accelerometer axes to mimic upside-down collar placement, circular time-shifts within windows to reduce sensitivity to exact temporal alignment, and the addition of low-amplitude Gaussian noise to approximate sensor noise.

#### 2.5.3. Feature Extraction

For each accelerometer window, a set of time and frequency features was extracted from the raw x-, y-. and z-axis signals, as well as from derived magnitude-based metrics. Extracted features included descriptive statistics (mean, standard deviation, minimum, maximum, median, 25–75% interquartile range), signal energy, entropy and cross-axis correlations. In addition, the vector of dynamic body acceleration (VeDBA) was calculated to capture movement intensity independent of sensor orientation. Dominant frequency components and zero-crossing rates were also extracted to improve discrimination between static and locomotor behaviors. The extracted features and their corresponding definitions/formulas are provided in Supplementary Table S1.

#### 2.5.4. Model Training and Validation

A random forest classifier [21] was trained using the extracted feature set. The random forest classifier was trained using 400 trees, a maximum tree depth of 10, and a minimum of 20 samples per leaf. A fixed random seed of 42 was used to ensure reproducibility. To account for inter-individual variability and assess model generalizability, a “Leave-One-Cow-Out” Group K-Fold cross-validation strategy was used, in which data from one individual were held out for testing while the model was trained on data from the remaining individuals. This procedure was repeated so that each individual served once as the test subject.

Class imbalances were adjusted using balanced class weight followed by an iterative process in which model parameters, feature sets, and preprocessing steps were evaluated and refined to improve classification performance. This included testing alternative parameter configurations and assessing their impact on classification metrics across cross-validation folds.

Model performance was evaluated for each cross-validation fold using classification metrics including accuracy, precision, recall, and F1-score. In this case, data from one cow is omitted and used as test data while the model is trained on the other individuals. Reported performance metrics reflect the best-performing model configuration identified through this iterative validation process with accuracy defined as the proportion of correctly classified windows across all behavioral classes. Precision was defined as the proportion of correctly predicted windows among all windows predicted for a given class, while recall represented the proportion of correctly predicted windows among all true windows belonging to that class. The F1-score was calculated as the harmonic means of precision and recall, providing a balanced measure of classification performance. Macro-averaged F1-scores were used to give equal weight to each behavioral class, regardless of differences in class frequency. Confusion matrices were generated for each cross-validation fold to evaluate patterns of misclassification between behavioral classes and to support interpretation of class-specific performance metrics. Confusion matrices for each test subject are provided in Supplementary Table S2 to allow detailed inspection of class-specific misclassification patterns without overloading the Section 3.

Following cross-validation and tuning of the model parameters, a final random forest model was trained using data from all individuals and saved for subsequent predictions on unlabeled accelerometer data. To improve the temporal consistency of predictions, model outputs were smoothed using a majority-vote filter across adjacent windows.

### 2.6. Statistical Analysis

Given the limited sample size and the exploratory nature of behavioral analyses, statistical analyses focused on within-individual comparisons and descriptive summaries rather than at the population level. All statistical analyses were conducted using R (version 4.5.2 [22]). We used the packages tidyverse (version 2.0.0; [23], ggthemes (version 5.2.0; [24]), sf (version 1.0-23; [25]), jsonlite (version 2.0.0; [26]) and data.table (version 1.18.0; [27]).

Outputs from the accelerometer-based behavior classification were used to derive individual-level behavioral metrics relevant to cattle activity and short-term responses to virtual fence warnings. Predicted behaviors were used to estimate individual-level daily time budgets, expressed as the proportion of time spent on the different behaviors over the study period.

To evaluate whether activity patterns differed in relation to proximity to the virtual fence, predicted behaviors were linked to GNSS positions. For each time window, the distance to the nearest virtual boundary was calculated, and based on this distance, observations were classified as either “near-boundary” or “interior”, using a fixed distance threshold (20 m). This threshold was chosen to account for GNSS positioning uncertainty and to provide a conservative buffer around the virtual fence boundary. Behavioral time budgets were then calculated for “near-boundary” and “interior” locations for each subject. Differences in the proportion of time spent in each behavior between the two spatial contexts were assessed using Wilcoxon signed-rank test, treating individuals as paired observations.

To assess potential short-term behavioral responses to warnings from the virtual fence system, event-based analyses were conducted using the Nofence warning logs along with accelerometer-derived movement intensity. This movement intensity was quantified using the VeDBA calculated while predicting behaviors. In the present study, the term “warning event” is used to refer collectively to logged Nofence events, including both auditory warnings and electrical impulses. During the study period, the Nofence logs contained 512 logged warning events across the seven animals, consisting of 486 auditory warnings and 26 electrical impulses. The number of logged events for each individual can be found in Table 2. For each warning event, VeDBA was extracted within a time window of 2 min before and 2 min after the warning. This time window were selected to provide a robust event-level comparison before and after warning events while reducing sensitivity to very brief fluctuations in the accelerometer signal. Changes in movement intensity were quantified as the difference between post-warning and pre-warning VeDBA. In addition, behavioral responses were evaluated by calculating changes in the proportion of windows classified as locomotion in the same event windows. The differences between post-warning and pre-warning were then analyzed using Wilcoxon signed-rank tests, with statistical significance defined as *p* < 0.05.

## 3. Results

### 3.1. Model Performance and Cross-Validation

After preprocessing and label refinement, a total of 31,447 overlapping accelerometer windows were retained for model training and evaluation. Across the three leave-one-cow-out test folds, the number of test windows was 10,225 for subject B, 10,153 for subject C, and 11,069 for subject E. Behavioral classes were unevenly represented, with grazing/feeding constituting the largest proportion of the data, followed by ruminating and lying, while standing and locomotion occurred less frequently. The random forest classifier demonstrated consistent performance across individuals when evaluating. Overall classification accuracy ranged from 0.83 to 0.90 across folds, with a mean accuracy of 0.87 (Table 3 and Supplementary Table S2). The mean macro-average F1-score across folds was 0.73, indicating balanced performance across behavioral classes.

Classification performance varied among behaviors (Table 4). Grazing/feeding showed consistently high precision and recall across all folds (F1-scores = 0.95). Ruminating and lying were also classified with high accuracy, with F1-scores ranging from 0.73 to 0.93. In contrast, standing and locomotion exhibit lower classification performance. Standing showed moderate recall but lower precision (F1-scores = 0.47–0.59), while locomotion had the lowest overall F1-scores (0.47–0.49). Confusion matrices revealed that misclassification primarily occurred between standing and lying or ruminating, and between locomotion and grazing/feeding or standing (Supplementary Table S2). Despite these challenges, performance patterns were consistent across cross-validation folds, suggesting that the model performs similarly across the labeled focal animals.

### 3.2. Behavioral Time Budgets

**Figure 2:**
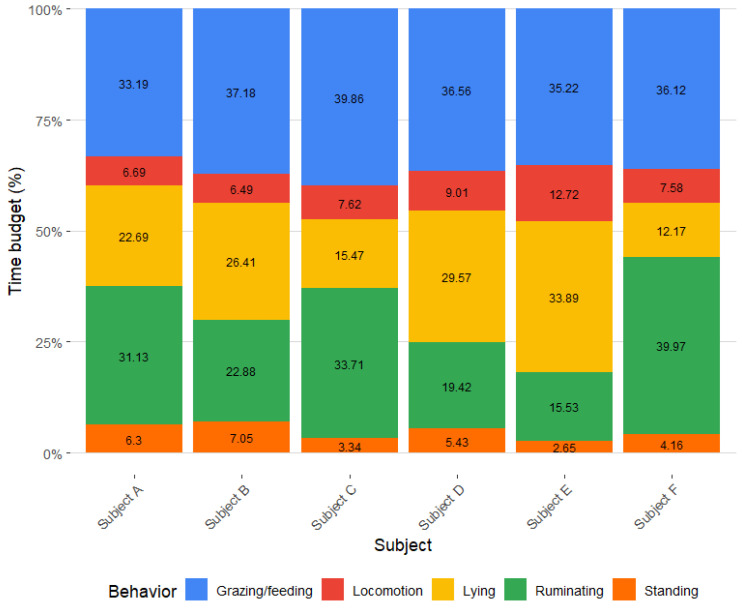
Overall behavioral time budgets predicted from accelerometer-based behavior classification for each individual across the study period. Values inside bars indicate the percentage of time predicted for each behavior. Grazing/feeding accounted for the largest proportion of time for most individuals, followed by ruminating and lying, while locomotion and standing constituted smaller proportions of the activity budget.

Predicted behaviors from the final trained model were used to estimate individual-level behavioral time budgets over the study period (Figure 2). Grazing/feeding accounted for the largest proportion of time for most individuals (33.19–39.86%), except for subject F, followed by ruminating (15.53–39.97%) and lying (12.17–33.89%). Locomotion (6.49–12.72%) and standing (2.65–7.05%) comprised smaller proportions of the total activity budget. Although individual variation in behavior allocation was observed, overall time budget patterns were similar across all subjects.

**Figure 3:**
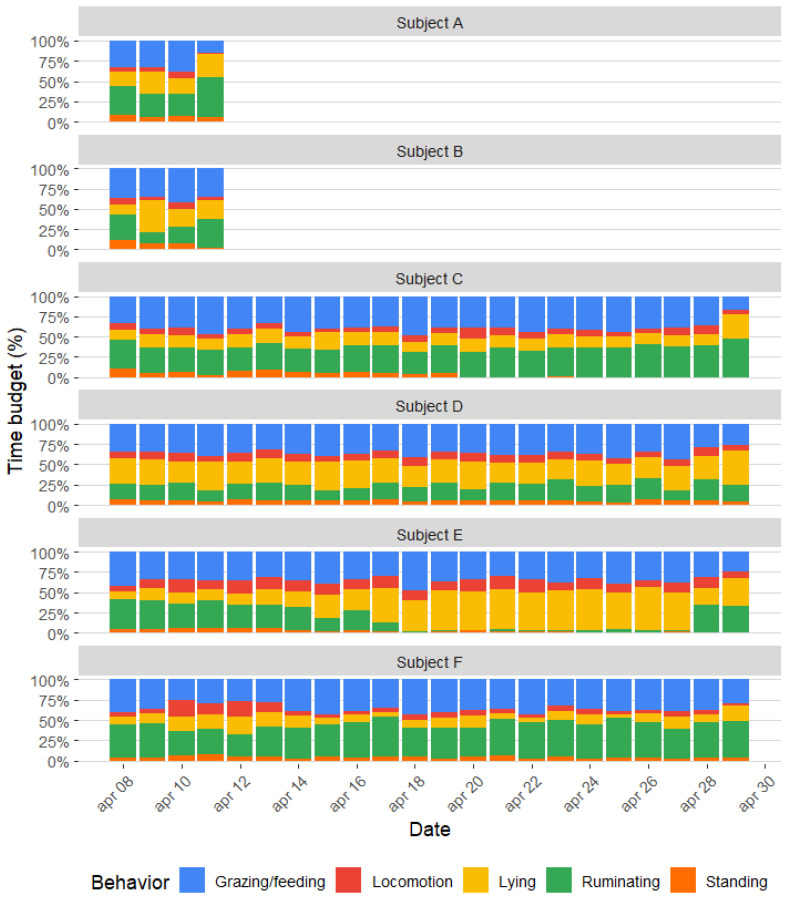
Daily behavioral time budgets for each individual across the study period. Values represent the proportion of time spent on each behavior. Bars represent the daily proportion of time spent on each predicted behavior.

To assess temporal variation in activity patterns across the study period, daily behavioral time budgets were calculated for each individual. The daily proportions of time spent in each behavior are shown in Figure 3. For most individuals, daily time budgets were relatively stable over time. In contrast, subject E showed a change in daily behavior allocation during the study period. From 15–27 April 2025, subject E exhibited a substantially higher proportion of time lying compared to other days, accompanied by a corresponding reduction in the behavior of ruminating. This shift was persistent across multiple consecutive days and was not observed in other individuals.

### 3.3. Behavioral Differences Relative to Proximity to the Virtual Fence

**Figure 4:**
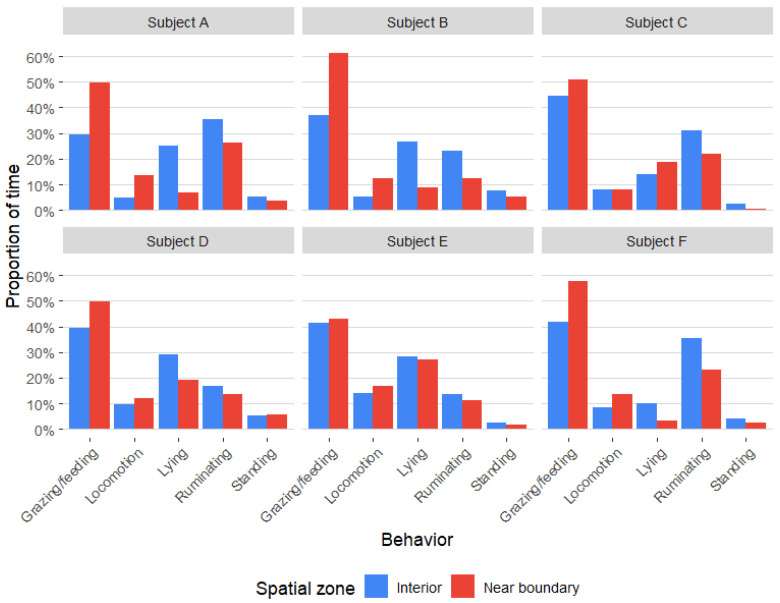
Comparison of behavioral time budgets between time windows classified as near-boundary (≤20 m from the virtual fence) and interior locations. Values represent proportions per individual.

Behavioral time budgets were compared between time windows classified as near-boundary (≤20 m from the virtual fence) and interior locations (Figure 4). Exploratory within-individual comparisons showed statistically significant differences in the proportion of time spend grazing/feeding and ruminating between near-boundary and interior locations. Grazing/feeding was higher near the virtual fence than in interior areas (*p* = 0.036), whereas ruminating occurred less frequently near the boundary (*p* = 0.036). Locomotion and standing both showed tendencies towards higher proportions near the fence, although these differences were not statistically significant (both *p* = 0.059). No significant differences were found for the behavior of lying (*p* = 0.093).

### 3.4. Short-Term Responses to Virtual Fence Warnings

Short-term responses to virtual fence warnings were evaluated using event-based analyses of movement intensity (VeDBA) and the predicted locomotion behavior.

**Figure 5:**
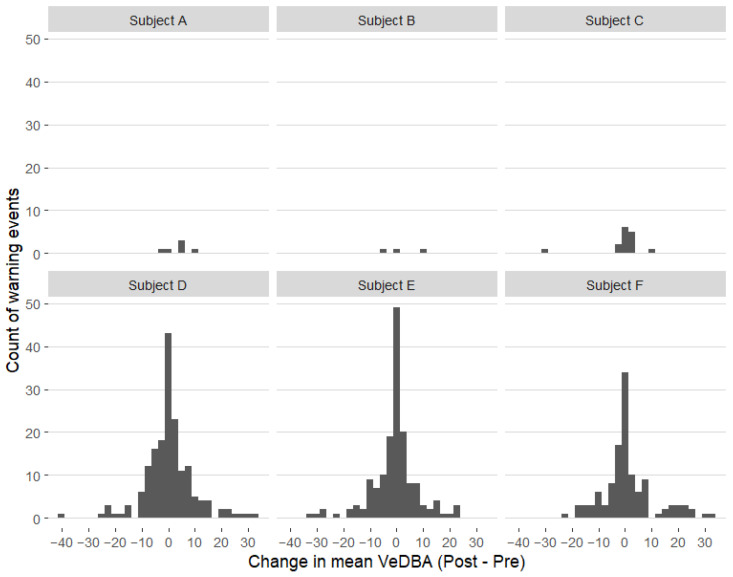
Distribution of changes in vector of dynamic body acceleration (VeDBA) calculated as the difference between post-warning and pre-warning values across all warning events.

The overall distribution of changes in mean VeDBA across all warning events is shown in Figure 5. The distribution is centered around zero for all individuals, indicating that warning events were not associated with a consistent increase or decrease in movement intensity. Both positive and negative changes were observed, showing variability in response among individual events.

**Figure 6:**
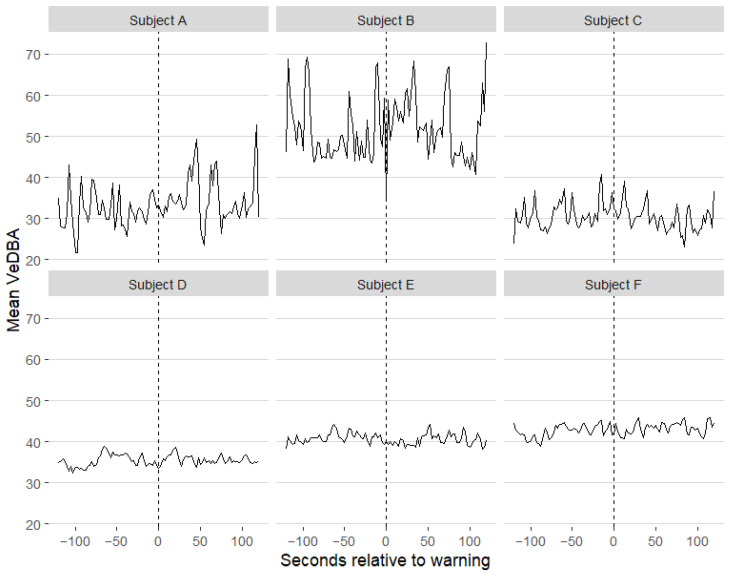
Event-level time series of the mean vector of dynamic body acceleration (VeDBA) for individual warning events. Each panel represents one individual, and each line represents the warning events aligned relative to the warning time (Seconds relative to warning = 0, marked with a dashed line). Mean VeDBA is shown across a 4 min analysis window spanning 120 s before and 120 s after each warning.

Event-level trajectories of mean VeDBA responses are shown in Figure 6, where individual warning events are aligned relative to the warning time (Seconds relative to warning = 0). The results show that mean VeDBA levels were generally stable within events, with no consistent changes observed around the time of the warning. Summary statistics for changes in mean VeDBA are presented in Table 5. The median change in VeDBA was close to zero (median = −0.019), and the mean changes were small (mean = 0.27). A Wilcoxon signed-rank test showed no significant overall difference between pre- and post-warning VeDBA values (*p* = 0.747).

**Figure 7:**
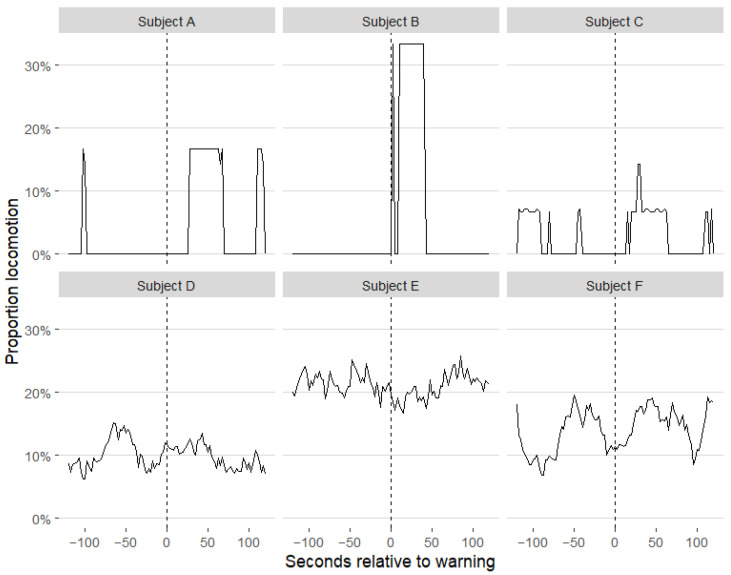
Event-level time series of the proportion of time windows classified as locomotion for individual warning events. Each panel represents one individual, and each line represents the warning events aligned relative to the warning time (Seconds relative to warning = 0, marked with a dashed line). The proportion of locomotion is shown across a 4 min analysis window spanning 120 s before and 120 s after each warning.

Behavioral responses were further evaluated by examining changes in the proportion of time windows classified as locomotion. Event-level changes in locomotion are shown in Figure 7. The figure shows that locomotion differed between individuals. However, within individuals, locomotion proportions were generally stable across the analysis windows. No consistent increase or decrease in locomotion was observed around the time of the warning. Short-lived increases and decreases in locomotion occurred in some events, but these were not temporally aligned with the warning and occurred both before and after warning events. Summary statistics for changes in locomotion are presented in Table 6. Changes in locomotion were generally small, with a median change of zero, a mean increase of 0.0044. No significant differences between pre- and post-warning locomotion were detected using a Wilcoxon signed-rank test (*p* = 0.365).

Visual inspection of Figure 5, Figure 6 and Figure 7 indicates that subjects A, B, and C differ from the remaining individuals in the apparent structure of event-level responses. These subjects were characterized by fewer warning events, resulting in different patterns in both VeDBA and locomotion time series. In contrast, individuals with a higher number of warning events showed smoother and more continuous trajectories across the analysis window.

Together, these results indicate that warning events were not associated with consistent short-term changes in either movement intensity (VeDBA) or the proportion of time spent in locomotion during the analyzed event windows.

## 4. Discussion

This study investigated cattle behavior in a virtual fencing system using collar-mounted accelerometers and random forest-based behavior classification. By combining predicted behaviors with GNSS data and warning logs from the virtual fence system, the study provides an assessment of both general activity patterns and short-term behavioral responses to fence warnings.

### 4.1. Accelerometer-Based Behavior Classification

The random forest model achieved high classification performance for the behaviors grazing/feeding, ruminating, and lying, which are key components of cattle time budgets and welfare assessments. This demonstrates that collar-mounted accelerometers are well-suited for capturing core behavioral states in cattle. The consistency of classification performance across the three cross-validation folds suggests that the model captured behavioral patterns shared among the labeled focal animals. However, as labeled observations were available from only three individuals, broader generalizability should be interpreted cautiously and require validation in larger datasets.

Lower classification performance for standing and locomotion is consistent with previous accelerometer-based studies [14,16], particularly when sensors are mounted on the neck rather than the limbs. This lower performance might be due to overlapping movement patterns with other behaviors when sensors are mounted to the neck. Ruminating occurs both standing and lying, and grazing can include smaller steps, which create an overlap between the behaviors. Despite these limitations, overall model performance was consistent across the labeled focal animals, indicating that the classification was sufficiently robust for exploratory prediction within the present dataset.

### 4.2. Behavioral Time Budgets and Individual Variation

Predicted behavioral time budgets showed that grazing/feeding dominates time budgets of most individuals, followed by ruminating and lying. In the present study grazing/feeding accounted for approximately 33–40% of the daily budget, ruminating for 15–40%, lying for 12–34%, locomotion for 6–13% and standing for 3–7%. These predicted values were broadly consistent with previously reported time budgets for grazing cattle, although rumination and resting-related behaviors showed greater individual variation.

Herbel and Nelson [28] reported that Hereford and Santa Gertrudis cows spent 37.2–42.8% of a 24 h period grazing, 30.8–31.0% ruminating, 7.5% standing, 9.9–10.1% lying and 6.5–12.1% walking. These values are comparable to the present study, particularly for grazing/feeding and locomotion, while the present study showed a wider range in rumination and lying.

Similarly, Cunningham et al. [4], using accelerometers, GPS and machine learning classification, reported daily time budgets of 7.6–9.9 h grazing, 5.5–7.8 h ruminating, 5.6–6.7 h stationary, and 1–3 h walking. Converted to percentages of a 24 h day, these correspond to approximately 31.7–41.3% grazing, 22.9–32.5% ruminating, 23.3–27.9% stationary, and 4.2–12.5% walking. The present study aligns closely with these ranges for grazing/feeding and locomotion, while rumination overlapped partly but was more variable among individuals. Because Cunningham et al. [4] grouped non-ruminating resting behaviors as stationary, whereas the present study separated lying and standing, direct comparison of resting behavior requires combining these categories. In the present study, lying and standing together ranged from approximately 16–37%, indicating broadly similar but more variable resting-time estimates.

Daily activity patterns are generally stable across individuals and throughout the study period, indicating consistent behavioral expression under virtual fencing management. One individual exhibits a prolonged increase in predicted lying behavior from 15–27 April 2025, accompanied by a reduction in predicted ruminating. Inspection of the raw accelerometer signals showed a clear shift in the signal pattern during the same period, indicating a likely change in sensor position or orientation. This suggests that the altered behavioral predictions were most likely related to sensor data rather than a true behavioral change. However, the animal was not examined specifically in response to this pattern beyond the farmer’s normal inspection routine, as the raw accelerometer data only became available after the removal of the sensor. This highlights a limitation of collar-mounted accelerometers, where changes in sensor position or orientation can influence behavior classification despite data augmentation strategies. Rather than reflecting a true behavioral shift, this case illustrates the importance of combining model outputs with raw signal inspection and direct inspection of the animal when unexpected patterns occur. It also emphasizes the need for robust orientation-invariant features when accelerometers are used for long-term monitoring.

### 4.3. Behavioral Patterns near the Virtual Fence

Behavioral differences between near-boundary and interior locations were subtle but detectable. Grazing/feeding were significantly more frequent near the virtual fence, while ruminating occurred significantly less near the boundary. These patterns suggest that cattle do not avoid the boundary once they habituate and are willing to exploit forage resources up to the virtual fence line. This finding is consistent with previous studies indicating that cattle can habituate to virtual fencing systems, with fewer electrical impulses over time [7,8]. In addition, previous work found no evidence of increased cortisol levels associated with virtual fencing [10]. The reduced occurrence of ruminating near the fence likely reflects the functional spatial organization of behavior rather than stress-related avoidance. The reduced occurrence of ruminating near the fence may therefore reflect functional spatial organization of behavior, where grazing and resting-related behaviors occur in different parts of the pasture.

An alternative explanation for the increased proportion of grazing observed near the virtual fence boundary may relate to spatial variation in forage availability or habitat type rather than behavioral responses to the fence itself. Forage biomass and quality were not measured directly along the virtual fence boundary in the present study, and it is therefore not possible to confirm whether forage resources were richer in these areas. However, spatial heterogeneity habitat use and preference has previously been documented in the same study area using GNSS-based virtual fencing data [9]. Increased grazing activity near the boundary could therefore reflect opportunistic exploitation of less-grazed vegetation or vegetation differences rather than attraction to, or avoidance of, the virtual fence. However, the absence of consistent short-term increases in movement intensity or locomotion following warning events, together with stable daily activity patterns, suggests that cattle were not persistently avoiding the boundary due to fear or stress once habituated to the system. Distinguishing between effects of habitat or forage distribution and behavioral responses to virtual fencing would require concurrent measurements of vegetation characteristics, which were beyond the scope of the present study.

### 4.4. Short-Term Responses to Virtual Fence Warnings

Event-based analyses revealed no consistent short-term changes in movement intensity (VeDBA) or locomotion following virtual fence warnings. Both distribution of changes in mean VeDBA and the event-level time series showed that responses varied among events but were centered around zero, with no systematic increase in activity immediately following warnings. The absence of a consistent response suggests that warning events, in a herd habituated to a virtual fence, do not show strong or uniform escape-like reactions [7,9]. From a behavioral perspective, this finding suggests that auditory warning events in habituated animals were not associated with measurable disruption of behavioral stability within the response variables analyzed here. Instead, cattle appear to respond in a context-dependent manner, with many warnings eliciting little or no measurable changes in movement intensity. Similarly, Verdon et al. [6] reported no high-aversion responses to virtual fencing warnings. Behaviors such as lunging, running, or head shaking were not observed following audio or electrical warnings during their study period. Instead, cattle most commonly turned and walked away from the boundary, or turned and resumed grazing.

Behavioral stability is the consistency of behavioral patterns over time and across individuals and is used as an indicator that animals are not experiencing persistent disturbance or stress. In the present study, behavioral stability was reflected by consistent daily activity budgets and the absence of prolonged changes in movement patterns following virtual fence warning events. Visual inspection of event-level trajectories further revealed differences between individuals. Subjects with fewer warning events, including individuals with limited accelerometer data coverage, showed sparser and more irregular response patterns. These differences likely reflect variation in data availability and event frequency rather than true behavioral differences.

The interpretation of predicted locomotion responses should be made cautiously because locomotion had relatively low class-specific performance in the behavior classification model. Therefore, the locomotion-based analysis should be viewed as complementary to the VeDBA analysis, which provides a direct measure of movement intensity independent of behavioral class prediction. It should also be noted that the use of mean VeDBA values within ±2 min windows may have reduced sensitivity to very short responses, such as brief head movements or short accelerations immediately after warnings.

### 4.5. Methodological Considerations and Limitations

Several methodological considerations should be acknowledged. First, accelerometer placement on the collar introduces sensitivity to sensor orientation, which can affect classification performance. Although data augmentation was applied to improve robustness to orientation changes, prolonged collar rotation may still influence predicted behavior. In this study, accelerometers could not be mounted in an orientation-stable position due to the structural design of the Nofence collar. To avoid interference with the collar’s functionality, the accelerometers were mounted on the rubber strap on the top of the collar. This placement increased the risk of sensor rotation over time as the collar moved on the animal’s neck. As a result, changes in sensor orientation could affect behavior classification, despite the use of data augmentation strategies to improve orientation robustness. Such orientation-related issues are therefore specific to the collar design used in this study and should not be interpreted as an inherent limitation of accelerometer-based behavior classification more generally.

Second, accelerometer data availability differed among individuals due to technical issues, resulting in shorter recording periods for some subjects. While this did not affect overall conclusions, it limits the number of warning events available for event-based analyses for certain subjects. Furthermore, the event-based analysis included both auditory warnings and electrical impulses as logged warning events, but these event types were not evaluated separately. Electrical impulses were relatively infrequent, with 26 impulse events compared with 486 auditory warnings during the study period. Because electrical stimulation is particularly relevant to welfare-related interpretation of virtual fencing systems, future studies should distinguish between audio-only warnings and electrical impulse events, especially during the initial learning phase when electrical impulses may occur more frequently.

Finally, the relatively small number of individuals restricts the generalizability of the findings. Although accelerometer-based behavioral analyses were conducted on six animals, labeled behavioral observations for model training and validation were available from only three focal animals. The predicted behavioral time budgets, spatial patterns, and warning-response analyses should therefore be interpreted as exploratory model-based estimates rather than definitive population-level conclusions. Despite these limitations, the consistency between behavioral time budgets, spatial analyses, and event-based results strengthens confidence in the overall conclusions.

### 4.6. Implications for Behavioral Monitoring Using Virtual Fencing

Taken together, the results suggest that accelerometer-based behavior classification may provide a useful, non-invasive tool for describing cattle behavior in virtual fencing systems. The absence of strong short-term behavioral responses to warnings, combined with stable daily activity patterns and only minor spatial differences near the boundary, suggests that no pronounced disruption of the measured behavioral patterns was detected in this habituated herd. More broadly, this study demonstrates how accelerometer data embedded in virtual fencing systems can be leveraged for continuous, non-invasive welfare monitoring. Future studies with larger sample sizes and longer monitoring periods could further explore individual differences in learning, adaptation, and welfare outcomes in virtual fencing systems, further evaluating the role of virtual fencing as both management and its implications for animal welfare [7,9].

## 5. Conclusions

This study demonstrates that collar-mounted tri-axial accelerometers, combined with supervised machine learning, can be used to characterize key behavioral patterns of free-ranging cattle managed with a virtual fencing system. The random forest model reliably classified core behaviors such as grazing/feeding, ruminating, and lying, enabling the estimation of individual behavioral time budgets under real-world grazing conditions. Behavioral patterns were generally stable across individuals and over time, with only minor spatial differences observed between areas near the virtual fence boundary and interior pasture locations. Importantly, short-term analyses revealed no consistent increases in movement intensity or locomotion following auditory warning events, indicating that habituated cattle do not exhibit uniform or pronounced behavioral disturbance in response to virtual fence warnings. While sensor orientation constraints related to collar design limited the classification of some behaviors, this study highlights the value of integrating accelerometer data, GNSS positioning, and virtual fence logs for fine-scale, animal-based behavioral assessment. Overall, within the limitations of the small labeled dataset and the habituated study herd, the findings suggest that virtual fencing was not associated with pronounced disruption of the measured behavioral patterns. The study also illustrates the potential of integrating accelerometer data, GNSS positioning, and virtual fence logs for animal-based behavioral monitoring in extensive grazing systems.

## Figures and Tables

**Table 1:** Ethogram used for behavioral observations during field data collection. Behaviors were recorded continuously for selected focal animals. The full ethogram reflects behaviors observed in the field, while the final classification column indicates which behaviors were retained, merged, or excluded before accelerometer-based machine learning analysis.

Behavior	Description	Used in Final Classification
Grazing/feeding	The animal is actively feeding with the head lowered	Yes
Ruminating	The animal is stationary and chewing cud. Can be while lying or standing	Yes
Walking	The animal is walking at a slow or moderate pace. Does not include small steps while grazing	Yes (merged with running into locomotion)
Running	Faster movement than walking	Yes (merged with walking into locomotion)
Standing	The animal is upright and stationary. Does not include ruminating while standing	Yes
Lying	The animal is lying down. Does not include ruminating while lying	Yes
Sudden movement/ reaction to fence	Includes all sudden movements that can occur as a reaction to warnings from a virtual fence. Such as: Head jerks, bolting and jumping	No (excluded)
Other	Includes all behaviors not in the other categories such as: Grooming, drinking, maintenance behavior (e.g., urinating) and social interaction with herd	No

**Table 2:** Number of logged Nofence warning events per animal during the study period. Events are separated into auditory warnings and electrical impulses. In total, 539 warning events were recorded, consisting of 510 auditory warnings and 29 electrical impulses.

Subject	Auditory Warnings	Electrical Impulses	Total Warning Events
Subject A	21	0	21
Subject B	14	1	15
Subject C	16	0	16
Subject D	170	3	173
Subject E	152	7	159
Subject F	113	15	128
Total	486	26	512

**Table 3:** Overall classification performance of the Random Forest model evaluated using “Leave-One-Cow-Out” cross-validation. Accuracy and macro-average F1-scores are reported for each test subject and as an overall mean.

Test Subject	Accuracy	Macro F1-Score
B	0.83	0.70
C	0.90	0.75
E	0.88	0.74
Mean	0.87	0.73

**Table 4:** Classification performance per behavioral class given as mean across cross-validation folds. Values represent averages across “Leave-One-Cow-Out” cross-validation folds. Individual performance and confusion matrix can be found in Supplementary Table S2.

Behavior	Precision	Recall	F1-Score
Grazing/feeding	0.96	0.95	0.95
Ruminating	0.90	0.91	0.90
Lying	0.83	0.82	0.81
Standing	0.48	0.56	0.51
Locomotion	0.50	0.47	0.48

**Table 5:** Summary statistics describing changes in the vector of dynamic body acceleration (VeDBA) calculated as the difference between post-warning and pre-warning values across all warning events.

VeDBA Event Effect					
Min	1st Quartile	Median	Mean	3rd Quartile	Max
−40.13	−3.41	−0.019	0.27	3.44	32.68

**Table 6:** Summary statistics describing changes in the proportion of time windows classified as locomotion, calculated as the difference between post-warning and pre-warning values across all warning events.

Locomotion Event Effect					
Min	1st Quartile	Median	Mean	3rd Quartile	Max
−0.89	0.00	0.00	0.0044	0.049	0.84

## Data Availability

Raw data supporting the findings of this study are available from the corresponding author upon reasonable request.

## Supplementary Materials

The following supporting information can be downloaded at: https://www.mdpi.com/article/10.3390/ani16132022/s1, Table S1: Definitions of accelerometer features extracted from each 5 s window; Table S2: Model performance and cross-validation.
