# Supplementary material for: Behavior Classification of Cattle in a Virtual Fencing System Using Tri-Axial Accelerometers and Machine Learning
Source: Animals (Basel). 2026 Jul 2;16(13):2022. doi: 10.3390/ani16132022 (PMC13360055; doi:10.3390/ani16132022)
Supplement: Supplementary file 1 [file animals-16-02022-s001.zip › animals-4316646-supplementary.pdf]

Supplementary Table S1. Definitions of accelerometer features extracted from each 5-s window

Table S1. Definitions of accelerometer-derived features used for behavioral classification. Features were extracted from each 5-s accelerometer window based on the raw x-, y-, and z-axis acceleration signals and derived magnitude-based metrics. Raw acceleration values were not used directly as model inputs but were used to calculate the statistical, correlation, entropy, frequency-domain, and zero-crossing features included in the random forest classifier.

Feature group	Feature name(s)	Formula / definition
Raw acceleration axes	x_i, y_i, z_i	Raw acceleration values recorded from the x-, y-, and z-axes within each 5-s window, where $i = 1, \ldots, n$. These values were not used directly as model inputs, but were used to calculate the derived statistical, magnitude-based, frequency-domain, and correlation features listed below.
Mean	x_mean, y_mean, z_mean, mag_mean, vedba_mean	$\bar{a} = \frac{1}{n}\sum_{i=1}^{n} a_i$
Minimum	x_min, y_min, z_min, mag_min, vedba_min	$\min(a_i)$
Maximum	x_max, y_max, z_max, mag_max, vedba_max	$\max(a_i)$
Standard deviation	x_std, y_std, z_std, mag_std, vedba_std	$SD = \sqrt{\frac{1}{n-1}\sum_{i=1}^{n}(a_i - \bar{a})^2}$
Median	x_median, y_median, z_median, mag_median, vedba_median	Median value of a_i within the window.
Interquartile range	x_iqr, y_iqr, z_iqr, mag_iqr, vedba_iqr	$IQR = Q_{75} - Q_{25}$
Signal energy	x_energy, y_energy, z_energy, mag_energy, vedba_energy	$Energy = \frac{1}{n}\sum_{i=1}^{n} a_i^2$
Magnitude	mag	$mag_i = \sqrt{x_i^2 + y_i^2 + z_i^2}$
Dynamic acceleration	dx, dy, dz	$dx_i = x_i - \bar{x}$, $dy_i = y_i - \bar{y}$, $dz_i = z_i - \bar{z}$
Vector dynamic body acceleration	vedba	$VeDBA_i = \sqrt{dx_i^2 + dy_i^2 + dz_i^2}$
Histogram-based entropy	mag_entropy, vedba_entropy	Entropy was calculated from a 20-bin histogram of a: $Entropy = -\sum h_j \log(h_j)$, where h_j represents non-zero histogram values.

Cross-axis correlation	xy_corr, xz_corr, yz_corr	Pearson correlation between pairs of raw acceleration axes. If either axis had zero standard deviation, the correlation was set to 0.
Dominant frequency	mag_dom_freq, vedba_dom_freq	The dominant frequency was identified as the frequency with the highest Fast Fourier Transform (FFT) amplitude after mean-centering the signal, within the frequency range $0 < f \leq 5$ Hz.
Zero-crossing rate	dx_zcr, dy_zcr, dz_zcr	$ZCR = \frac{1}{n-1}\sum_{i=1}^{n-1} I(a_i * a_{i+1} < 0)$ where I is an indicator function.

Supplementary Tables S2. Model performance and cross-validation

Subject B

Confusion matrix

True \ Predicted	Grazing/feeding	Lying	Ruminating	Standing	Locomotion
**Grazing/feeding**	3949	56	20	84	53
**Lying**	14	1707	511	427	0
**Ruminating**	3	34	2394	50	0
**Standing**	68	192	49	395	11
**Locomotion**	90	6	4	21	87

Classification performance per behavioral class

	Precision	Recall	f1-score	Support
**Grazing/feeding**	0.96	0.95	0.95	4162
**Lying**	0.86	0.64	0.73	2659
**Ruminating**	0.80	0.96	0.88	2481
**Standing**	0.40	0.55	0.47	715
**Locomotion**	0.58	0.42	0.48	208
**accuracy**			0.83	10225
**macro avg**	0.72	0.71	0.70	10225
**weighted avg**	0.85	0.83	0.83	10225

Subject C

Confusion matrix

True \ Predicted	Grazing/feeding	Lying	Ruminating	Standing	Locomotion
**Grazing/feeding**	5396	0	1	175	192
**Lying**	17	1464	86	103	21
**Ruminating**	17	36	1844	51	9
**Standing**	53	31	75	234	25
**Locomotion**	135	0	0	13	175

Classification performance per behavioral class

	Precision	Recall	f1-score	support
**Grazing/feeding**	0.96	0.94	0.95	5764

**Lying**	0.96	0.87	0.91	1691
**Ruminating**	0.92	0.94	0.93	1957
**Standing**	0.41	0.56	0.47	418
**Locomotion**	0.41	0.54	0.47	323
**Accuracy**			0.90	10153
**macro avg**	0.73	0.77	0.75	10153
**weighted avg**	0.91	0.90	0.90	10153

Subject E

Confusion matrix

True \ Predicted	Grazing/feeding	Lying	Ruminating	Standing	Locomotion
**Grazing/feeding**	5471	0	23	55	204
**Lying**	0	1120	7	39	0
**Ruminating**	13	417	2586	62	0
**Standing**	30	140	43	306	27
**Locomotion**	258	0	3	22	243

Classification performance per behavioral class

	Precision	Recall	f1-score	support
**Grazing/feeding**	0.95	0.95	0.95	5753
**Lying**	0.67	0.96	0.79	1166
**Ruminating**	0.97	0.84	0.90	3078
**Standing**	0.63	0.56	0.59	546
**Locomotion**	0.51	0.46	0.49	526
**accuracy**			0.88	11069
**macro avg**	0.75	0.75	0.74	11069
**weighted avg**	0.89	0.88	0.88	11069
